# Distinctive Inflammatory Traits of Parvovirus B19 Infection Associated with Persisting Autoimmunity

**DOI:** 10.3390/v18070774

**Published:** 2026-07-14

**Authors:** Anna Negri, Maria Chiara Gerardi, Gabriele D. Gallina, Luca Moroni, Antonella Adinolfi, Mona-Rita Yacoub, Nicola Boffini, Marco Lanzillotta, Annalaura Fasiello, Claudia Cordini, Enrica P. Bozzolo, Marco Matucci-Cerinic, Oscar Massimiliano Epis, Lorenzo Dagna, Giuseppe A. Ramirez

**Affiliations:** 1 Faculty of Medicine, Università Vita-Salute San Raffaele, via Olgettina 58, 20132 Milan, Italy; a.negri2@studenti.unisr.it (A.N.); matuccicerinic.marco@hsr.it (M.M.-C.); ramirez.giuseppealvise@hsr.it (G.A.R.); 2 Unit of Immunology, Rheumatology, Allergy and Rare Diseases, IRCCS Ospedale San Raffaele, via Olgettina 60, 20132 Milan, Italy; moroni.luca@hsr.it (L.M.); lanzillotta.marco@hsr.it (M.L.); bozzolo.enrica@hsr.it (E.P.B.); 3 Unit of Rheumatology, IRCCS Ospedale Niguarda, Piazza dell’Ospedale Maggiore, 3, 20162 Milan, Italyclaudia.cordini@ospedaleniguarda.it (C.C.);; 4 Advanced Multidisciplinary Center for Asthma, Food and Drug Allergies, IRCCS Ospedale San Raffaele, via Olgettina 60, 20132 Milan, Italy; 5 Inflammation Fibrosis and Ageing Initiative (INFLAGE), Division of Genetics and Cell Biology, IRCCS Ospedale San Raffaele, via Olgettina 60, 20132 Milan, Italy

**Keywords:** parvovirus B19, autoimmune diseases, epidemiology, outbreak

## Abstract

Background: Adult parvovirus B19 (B19V) infection occurs with cyclic outbreaks and can be associated with inflammatory manifestations mimicking or heralding the onset of systemic autoimmune diseases. Little is known about clinical traits distinguishing patients with transient vs. persisting manifestations. Methods: Leveraging data from the 2024 B19V infection outbreak, we set up a retrospective, multicentre, observational study investigating the epidemiology and clinical phenotypes of patients with B19V infection presenting to tertiary care for immune-mediated manifestations, including patients with pre-existing autoimmunity. Results: A total of 39 B19V infections were identified, yielding an annual incidence of 1.78 cases/1000 patients. Autoimmune rheumatic diseases were pre-existing in 7/39 and newly diagnosed in 5/39. One patient was hospitalised. Arthritis was more frequent in new-onset (89%) than in pre-existing (14%; *p* = 0.010) or no autoimmunity (19%; *p* < 0.001). Anaemia was not found in patients with pre-existing autoimmunity in contrast to those with new autoimmune disease diagnoses (40%; *p* = 0.039) or transient manifestations (52%; *p* = 0.013). Skin manifestations were numerically less frequent in this latter group (56%) than in patients with pre-existing (100%) or newly onset autoimmunity (80%). Autoantibodies were detected in 44% of cases and were more frequent in patients with constitutional symptoms but were not confirmed in the long term in most cases. Glucocorticoids were more frequently employed in patients with new-onset rather than pre-existing autoimmune diseases (*p* = 0.006) or without chronic autoimmunity (*p* = 0.027). Conclusion: B19V-associated new-onset autoimmunity may occur in one in six cases observed in tertiary immunology care, show distinct clinical features, and require significant immunosuppression.

## 1. Introduction

Parvovirus B19 (B19V) is a small, non-enveloped, single-stranded DNA virus of the Parvoviridae family [1]. It primarily infects erythroid progenitor cells in the bone marrow but can also affect other organs/tissues such as the heart, the liver, and the synovium [2,3,4,5]. B19V is common worldwide and better known as the etiological agent of erythema infectiosum (“fifth disease”) in children. In adults, B19V has also been associated with a broad spectrum of clinical manifestations, such as transient aplastic crises, persistent anaemia, pregnancy complications (foetal anaemia, foetal hydrops, miscarriage and intrauterine foetal death) and acute or chronic arthropathy [1,2,3,4,5]. Epidemiologically, B19V infections peak annually in winter and spring and show cyclic major outbreaks every 3–4 years. Viral transmission usually occurs by the respiratory route [6].

In most European Union/European Economical Area (EU/EEA) Member States, B19V infection is not systematically monitored, preventing accurate estimations of B19V infection trends across the continent. Nonetheless, in May 2024, the European Centre for Disease Control (ECDC) reported a notable increase in B19V infections across many European countries. This phenomenon was thought to be a consequence of reduced virus circulation during the COVID-19 pandemic, which had potentially reduced population immunity and increased the risk of rebound outbreaks, possibly with unusual seasonality [7,8]. In Italy, where B19V surveillance is not mandated nationally, data from plasma donation screening revealed a major peak in B19V infection in March 2023, with a ratio of 59.4 B19V-positive units per 100,000 donations [9]. This increase was particularly significant in northern Italy, as documented in studies from the metropolitan area of Bologna [10] and from the Region of Veneto [11].

In adults, B19V infection may mimic systemic autoimmune rheumatic diseases. In particular, joint involvement occurs in 50% of adult cases, with a higher frequency in women. It usually presents as a small-joint hand symmetric polyarthritis and less frequently as arthritis affecting ankles, knees or wrists [2,6,12,13,14]. Joint symptoms are often accompanied by rash, purpura, fever, and laboratory abnormalities, such as transient autoantibody positivity and hypocomplementemia [15,16,17]. Recent studies demonstrated that this autoimmune-like presentation can often fulfil the classification criteria for rheumatic diseases such as rheumatoid arthritis (RA) and systemic lupus erythematosus (SLE), leading to misdiagnosis and unnecessary treatment [15,18]. Indeed, infections are generally associated with exacerbation and/or triggering of clinically overt autoimmunity in predisposed individuals [19,20,21,22]. Conversely, symptoms of infections are often mimickers of autoimmune manifestations both in patients with established autoimmunity [23] and in healthy individuals [24]. While infection-associated inflammatory symptoms usually resolve within days or weeks and are generally not associated with tissue damage [6,25], infection-triggered persisting autoimmunity may lead to chronic morbidity.

To date, limited evidence exists with regard to the potential distinctive features of subjects who present with infections and eventually develop chronic autoimmunity [26]. This evidence is particularly scarce for B19V, also in light of its elusive and fluctuating epidemiological features. To address this issue, after observing a potential rise in B19V infection cases referred to our clinical units for inflammatory manifestations, we decided to systematically investigate all B19V infection cases recorded over the course of one year and dissect potential clinical indicators of progression to overt autoimmune disorders.

## 2. Materials and Methods

### 2.1. General Design

This was a retrospective, multicentre, observational study including consecutive patients with evidence of B19V infection referred to the Allergy and Rheumatology Clinics of San Raffaele Hospital and of ASST Grande Ospedale Metropolitano Niguarda of Milan between 1 January 2024 and 31 December 2024. All enrolled subjects provided written informed consent for study participation under the Panimmuno Research Protocol (approved by the San Raffaele Ethics Committee on 8 March 2018, ref. 22/INT/2018) and the GISEA EARLY protocol (approved by Niguarda Ethics Committee on 24 June 2016, ref. 256-062016), both conforming to the Declaration of Helsinki.

Patients were included when they had a recent history of B19V infection, supported serologically (either by detection of serum IgM or evidence IgG or by detection B19V-DNA) or epidemiologically, based on a history of recent contacts with an infected individual associated with typical clinical manifestations. Administrative data regarding the number of outpatient accesses during the observation timeframe of interest were available for one of the two participating centres (San Raffaele Hospital) and were used to estimate the annual incidence of B19V-infected patients accessing an allergy/rheumatology clinic in 2024. All patients were evaluated at a baseline time, coinciding with the first clinical evaluation after B19V infection. Patients with short-term, self-resolving inflammatory and/or autoimmune manifestations did not have further evaluations by clinical practice. Patients with persisting symptoms were further evaluated during the following months and data regarding their clinical course recorded in our study.

### 2.2. Immunological Parameters

We categorised all enrolled individuals into three categories: (1) patients with pre-existing autoimmune rheumatic diseases; (2) patients who developed an autoimmune rheumatic disease after B19V infection; (3) patients with B19V infections with only transient inflammatory manifestations, including (a) clinical and/or serological signs of autoimmunity and (b) post-viral inflammatory syndromes with no signs of autoimmunity. Only patients whose diagnosis was confirmed after at least one follow-up visit after the first evaluation were included in Group 2. Patients with known or newly onset autoimmune diseases were classified according to standardised criteria. Specifically, we used the 2019 American College of Rheumatology (ACR)/European Alliance of Associations for Rheumatology (EULAR) classification criteria for SLE) [27], the 2010 ACR/EULAR classification criteria for RA [28], the 2009 Assessment of SpondyloArthritis (SpA) international Society (ASAS) criteria for axial spondyloarthritis [29] and the 2011 ASAS criteria for peripheral SpA [30], the 2023 ACR/EULAR classification criteria for antiphospholipid syndrome [31], and the 2015 revised Jones criteria (American Heart Association revision) for rheumatic fever [32]. Undifferentiated connective tissue disease (UCTD) was classified according to the criteria by Mosca et al. [33]. Persisting arthritis not meeting the criteria for specific disorders was classified as undifferentiated arthritis [34]. Clinical diagnostic criteria for Hashimoto’s thyroiditis [35] were also applied.

Baseline clinical data encompassed patient demographics, past medical history, and chronic treatments. Chronic comorbidities included hypertension, cardiovascular disorders, metabolic/endocrine disorders, diabetes, obesity or overweight, hypothyroidism, osteoporosis, obstructive pulmonary diseases, upper airway disorders, renal disorders, gastrointestinal disorders, neurological disorders, uveitis or other ocular diseases, endometriosis, any type of cancer, and receipt of organ transplants. Besides autoimmune comorbidities, we also recorded immunodeficiency, allergic disorders and mastocytosis as potential coexisting conditions. In patients with pre-B19V autoimmunity, we recorded disease extent and duration and the presence of disease-associated serological abnormalities. We also recorded whether patients were or were not in remission according to the clinician’s general impression.

### 2.3. B19V Infection Features

Specific data regarding B19V infection presentation included the occurrence of fever and/or other constitutional symptoms [36], musculoskeletal involvement (including arthralgia and arthritis), cutaneous manifestations (including rash, purpura and livedo), and neurological manifestations (in particular peripheral nervous system involvement). We also collected a set of laboratory parameters tested as part of routine clinical practice. These variables included complete blood count, serum protein electrophoresis abnormalities, complement consumption, liver enzyme alterations, and the appearance of previously unknown autoantibodies, including antinuclear antibodies (ANA), anti-double-stranded DNA antibodies (anti-dsDNA), antineutrophil cytoplasmic antibodies (ANCA), antiphospholipid antibodies (aPL), and rheumatoid factor (RF).

### 2.4. Statistical Analyses

Data were elaborated using Microsoft Excel^®^ 2019 and SPSS^®^ software version 21.0 (IBM Corp., Armonk, NY, USA). The chi-square test was used to compare categorical variables among groups. Fisher’s exact correction was applied as appropriate. The Mann–Whitney U test and Kruskall–Wallis test were used to test differences in non-normally distributed continuous variables between two or more groups. Data are expressed as percentages (categorical variables) or median (interquartile range, IQR: quantitative variables) unless otherwise specified.

## 3. Results

### 3.1. Demographics and Epidemiological Data

A total of 39 patients were enrolled. Administrative data were available for one of the two centres and showed that out of 11,799 patients accessing Allergy and Rheumatology Clinics between 1 January 2024 and 31 December 2024, 21 presented with B19V infection, yielding an annual proportion of 1.78 cases per 1000 patients. Out of the total number of enrolled subjects, 30 were women (77%), and the median age was 42 (36–46) years. Some 9 patients (23%) had a known diagnosis of autoimmunity, including 7 patients with autoimmune rheumatic disorders, while 30 had no prior history of autoimmunity at baseline. B19V infection diagnosis was made on an epidemiological basis in 7 patients (18%), on serological analysis in 30 (77%), and on viral DNA detection in 2 (5%). A sensitivity analysis to test for potential biases introduced by including patients with epidemiological diagnoses only did not reveal significant differences in demographics and clinical features with patients receiving virological diagnoses (Table S1). Serological testing showed IgM positivity in 25 patients (64%) and IgG positivity in 4 (10%; Table 1).

### 3.2. Clinical Presentation at Baseline

Some 22 patients (56%) developed constitutional symptoms, including fever in 19 (49%). Arthralgia was the most common clinical manifestation, occurring in 31 patients (80%) and mainly involving the hands. Ten patients (26%) also developed arthritis (Figure S1). Cutaneous involvement was also reported in 26 patients (67%), including rash (44%), purpura (13%), livedo (5%), pruritus and ecchymosis. Neurological manifestations occurred in five patients (13%), mainly as dysesthesia, all resolving by the last follow-up visit. One patient (3%) also developed serositis in the form of pleural effusion. Laboratory tests showed anaemia in 16 patients (41%), leukopenia in 7 (18%, including 4 cases of lymphopenia and 2 cases of neutropenia), and thrombocytopenia in 1 (5%). Hypergammaglobulinemia was detected in nine patients (23%) and elevated liver enzymes in seven (18%). Moreover, autoantibody positivity was detected in 17 patients (44%) after 11 (0–46) days from symptom onset. Autoantibodies included ANA in 11 cases (28%), anti-dsDNA in 3 cases (8%), RF in 3 cases (8%), ANCA in 2 cases (5%), and aPL in 2 cases (5%). Cryoglobulins were positive in three patients (8%), without clinical manifestations of cryoglobulinemia. Low complement was observed in nine patients (23%; Table 2).

**Figure 1:**
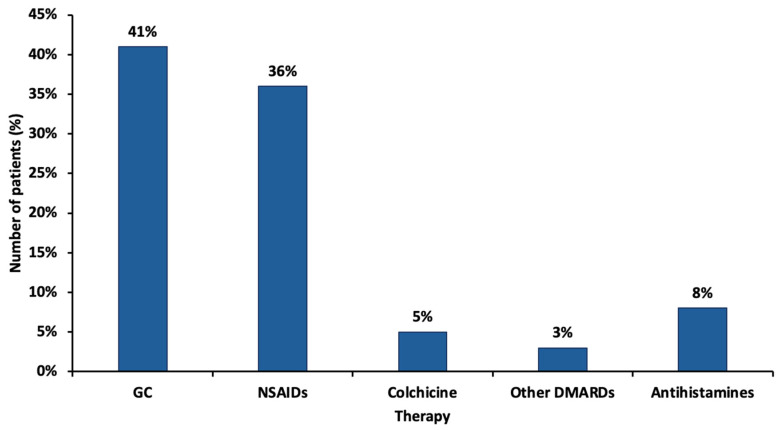
Pharmacological treatments in the study population. The bar chart shows the frequency of the most frequent treatment used for symptom relief and for contrasting inflammatory manifestations due to parvovirus B19 infection in the study cohort. Abbreviations: GC: glucocorticoids, DMARDs: Disease-Modifying Antirheumatic Drugs, NSAIDs: Non-steroidal Anti-Inflammatory Drugs.

Only one patient required hospitalisation for fever, widespread arthromyalgia, and pleural effusion. Twenty-seven patients (69%) received pharmacological treatment for a median (IQR) time of 28 (10–40) days. Sixteen patients (41%) were treated with glucocorticoids with a median prednisone-equivalent starting dose of 25 mg/day and a median (IQR) duration of treatment of 30 (27–41) days. Fourteen subjects (36%) received Non-steroidal Anti-Inflammatory Drugs (NSAIDs). Other treatments included colchicine (5%), other classical Disease-Modifying Antirheumatic Drugs (DMARDs; 3%), and antihistamines (8%). No patient needed biotechnological DMARDs (Figure 1).

### 3.3. Follow-Up and New-Onset Autoimmune Diseases

Follow-up serological data were available for 11/17 patients with autoantibodies at baseline: only 2/11 retained positive autoantibodies after 5 (4–8) months from initial assessment, with one showing multiple persisting autoantibodies (see below).

A total of 5 (13%) patients with persisting autoimmune symptoms/signs after 1 (1–4) months were diagnosed with an autoimmune disease and were compared with the 7 patients with pre-existing autoimmunity and with the remaining 27 patients with self-resolving inflammation. New autoimmune disease diagnoses were further confirmed after additional 11 (4–13) months. Two patients (5%) developed a form of seronegative arthritis which could not be classified as RA, axial or peripheral SpA, arthritis in the setting of CTDs, or other definite forms of arthritis. While both patients had persisting arthritis at the follow-up visit, one of them had transiently positive ANA, which was eventually not confirmed. Two (5%) patients developed SpA, with one showing transient ANA positivity and one transiently positive RF. One patient was diagnosed with UCTD and maintained positive ANA, RF, and low complement across the follow-up.

### 3.4. Distinctive Traits of Patients with New-Onset or Pre-Existing Autoimmune Diseases

Arthritis was detected in all groups but was significantly more frequent in new-onset autoimmune disorders (80%) than in patients with pre-existing autoimmunity (14%; *p* = 0.010) or no chronic autoimmunity (19%; *p* < 0.001). One case of serositis was detected among patients with new diagnoses of autoimmune diseases, while no cases were found in the remaining groups. Skin manifestations were numerically more prevalent in patients with new-onset (80%) or pre-existing (100%) autoimmune diseases than in patients with no chronic autoimmunity (56%; *p* = 0.067). Anaemia was relatively frequent in patients with new-onset autoimmune diseases (40%) and in patients with no chronic autoimmunity (52%), while no cases of new-onset anaemia were detected among patients with pre-existing autoimmune diseases (*p* = 0.039; and *p* = 0.013, respectively). Patients with a new diagnosis of an autoimmune disease required glucocorticoids more frequently than patients with a previous autoimmune disease (*p* = 0.006) and without chronic autoimmunity (*p* = 0.027). No differences were observed in terms of prednisone-equivalent dose and duration of glucocorticoid treatments among groups. No patient with autoimmune disorders (either pre-existing or newly diagnosed) required further glucocorticoid treatment during follow-up.

We also observed that baseline autoantibodies were found in patients with more pronounced constitutional symptoms, (OR = 15.9 *p* < 0.001), especially in the case of ANA (OR= 4.9 *p* = 0.044). Among patients with purpura (n = 5), a trend toward the development of autoantibodies was observed, but only the association with ANCA reached statistical significance (OR = 22, *p* = 0.004).

## 4. Discussion

In this study, we observed that B19V infections presenting with inflammatory symptoms were frequently referred to tertiary care for immunological consultations during the course of 2024, with 16% of patients with no history of autoimmunity developing a new autoimmune disorder. The most frequent clinical features were constitutional symptoms, musculoskeletal and mucocutaneous manifestations, and haematological abnormalities. Autoantibodies were frequently detected, but only a very small fraction of individuals were persistently positive during follow-up. The course of B19V infection and sequelae was benign in most cases, with only one patient requiring hospitalisation. Arthritis, anaemia, possibly serositis and increased use of glucocorticoids selectively identified patients with new-onset autoimmunity, while skin manifestations were relatively more frequent in patients who had or received a diagnosis of an autoimmune disease.

In rheumatology, viral infections are recognised as possible triggers of autoimmunity in susceptible patients and as causes of exacerbation in patients already affected by an autoimmune rheumatic disease [19,37,38]. Rubella virus, Human T-lymphotropic virus 1 (HTLV-1), hepatitis-B virus, and Epstein–Barr virus (EBV) have been associated with RA [39,40]. HTLV-1 and Hepatitis C virus have been linked to Sjogren syndrome [40]. EBV, along with a variety of viruses including retroviruses and cytomegalovirus (CMV), has been implicated in triggering SLE. Moreover, infections caused by Herpesviridae (Varicella Zoster Virus, Herpes Simplex Virus, EBV, CMV), Mycobacterium tuberculosis, Mycobacterium leprae, and SARS-CoV-2 have been associated with SLE flares [19,23,40]. Limited epidemiological data are available regarding the baseline prevalence of B19V in the general population and its impact on autoimmune disorders [41]. When compared to indirect estimates from plasma donors [9], our data seem to indicate that during B19V outbreaks (as in 2023–2024 [42]), further enrichment of symptomatic cases could possibly have been observed in an immunology tertiary care setting. In this regard, the association between B19V infection and clinical manifestations mimicking autoimmune rheumatic diseases has been described in the literature [6,15,17,43,44,45,46,47,48]. Adding to previous knowledge highlighting the risk of misdiagnosis of autoimmune disorders in short-term post-B19V infection follow-up, our study suggests that a subset of subjects could develop self-sustaining autoimmunity triggered by B19V and might possibly show specific clinical at-risk profiles at the time of symptom presentation. Our results further corroborate the importance of persisting clinical manifestations rather than serological alterations in supporting the diagnosis of new-onset autoimmune diseases.

Our data also contribute to refining current understanding of the clinical and prognostic characteristics of post-viral syndromes. While more extensive data have been acquired on long COVID-19 syndrome in recent years [49], the boundaries of long-term B19V-associated manifestations remain less defined [48,50]. In our population, the most frequent manifestation was joint involvement (82%), including arthralgia (80%) and arthritis (26%). Consistently, asymmetric small-joint polyarthralgia/arthritis typically affecting middle-aged subjects is reported as a typical presentation of B19V infection in adults in the literature [6,12,13]. In our cohort, arthritis was relatively more frequent in patients with new-onset autoimmunity compared to the other groups, suggesting that it could represent a clinical hint for identifying patients at higher risk of developing infection-triggered chronic autoimmune diseases [19]. Several pathogenic mechanisms have been proposed to explain the development of arthritis during B19V infection, including activation of monocytes and lymphocytes within the synovium, antibody-dependent enhancement (ADE) of virus pathogenicity leading to local inflammation, and activation of synoviocytes by viral-associated phospholipase A2. Interestingly, B19V-related joint manifestations have been associated with the detection of leukocyte-shuttled viral particles within affected joints, which mirrors mechanisms identified in the setting of established rheumatic disorders such as axial SpA [2,51,52,53].

Skin manifestations were also frequent in our cohort (67%) and represent the most obvious clinical features of B19V infection in childhood, though also being consistently part of the adult presentation frame [54]. B19V is thought to infect endothelial cells through ADE, impairing local vasomotor control and/or causing overt small-vessel vasculitis [55,56,57]. In this regard, direct cytotoxicity may synergise with autoantibodies. Consistently, in our cohort, ANCA was associated with the development of purpura [55,56]. In addition to endothelial cell infection, B19V-associated skin manifestations are usually accompanied by disruption of collagen architecture and histiocyte/lymphocyte infiltration of dermal interstitial spaces along with interface dermatitis, thus overlapping with findings observed in systemic autoimmune disorders [56]. Consistently, skin manifestations were more frequent in patients with definite autoimmune disorders than in patients with transient inflammatory B19V symptoms in our cohort.

Anaemia constitutes a hallmark of B19V infection due to erythroid progenitor cell susceptibility to B19V [58]. In our series, four out of ten subjects developed anaemia, with the notable exception of patients with pre-existing autoimmunity. This finding is in contrast with the observation of enhanced vulnerability to severe anaemia in patients with pre-existing diseases characterized by abnormal haematopoiesis [17]. Ongoing immunomodulant/immunosuppressive treatments in patients with pre-existing autoimmunity may confer protection from B19V-related anaemia and to other potentially severe B19V-related manifestations. Consistently, we observed higher use of corticosteroids in patients with new-onset autoimmune disorders.

The development of autoantibodies constitutes an additional feature consistently reported in association with B19V in the literature [16,42,46,47,48,50,59]. Polyclonal activation of the adaptive immune response represents a simple mechanism potentially accounting for this finding. However, experimental evidence also suggests the direct involvement of B19V in promoting the disruption of immunological tolerance. Virus-induced apoptosis may increase autoantigen load and facilitate autoimmunity. B19V VP1 protein can further contribute to this aspect by aberrantly cleaving cell phospholipids [43]. Furthermore, antibodies targeting B19 components are able to cross-react with autoantigens, suggesting a role for molecular mimicry in B19V-related autoimmunity [60]. In our cohort, we observed that 44% of the subjects tested positive for autoantibodies at baseline. Furthermore, we found that the development of autoantibodies was correlated with constitutional symptoms, mirroring similar observations in the setting of COVID-19 [61] and possibly suggesting a linear correlation between the intensity of the immune drive incited by B19V and the degree of loss of tolerance towards self-antigens.

Taken together, our data reinforce the notion that B19V can be implicated in a quite heterogeneous spectrum of inflammatory manifestations, which can evolve in self-sustained chronic autoimmunity and/or cause disease flares in patients with pre-existing autoimmunity. Our results also suggest that specific clinical phenotypes might help to predict which patients will eventually develop autoimmunity or self-limiting manifestations. These considerations should, however, be critically interpreted, taking into consideration a set of intrinsic limitations of our study. Despite taking advantage of the contribution of two tertiary referral centres for the treatment of immune-mediated diseases, this study still included only a relatively small number of patients, therefore restricting the robustness and generalisability of its conclusions. Furthermore, the small sample size could have limited our ability to detect rarer manifestations (such as myocarditis) and assess their prognostic role. Specifically, due to the very low number of patients developing established new autoimmune diseases, our results in this regard must be interpreted more as hypothesis-generating than as conclusive. In addition, while more frequent use of glucocorticoids in patients with new-onset autoimmune diseases might retrospectively corroborate the hypothesis that patients in this subgroup differed from those with transient inflammation due to the need for more aggressive treatment, the fact that glucocorticoids and NSAIDs were used in a non-negligible proportion of patients in the whole cohort could also have altered the natural history of patients with milder susceptibility and affected our epidemiological estimates by reducing the number of new diagnoses. Second, our study lacks experimental evidence, which could have shed light on the pathogenic mechanisms driving distinct B19V-related phenotypes in patients with autoimmune diseases. Third, the retrospective multicentre design of the study could have introduced additional biases due to missing data (for example, about the pre-B19V serological status of patients who eventually developed clinically overt autoimmunity) and heterogeneity in diagnostic approaches, clinical management, and data collection. In addition, since the study was restricted to tertiary immunological referral centres, our epidemiological estimates of B19-related new autoimmune disease incidence may not entirely overlap with trends observed in the general population. Therefore, larger, long-term population-based studies are required to confirm and corroborate our data.

## 5. Conclusions

The 2024 B19V infection outbreak caused a possible surge in patients presenting to tertiary care for inflammatory manifestations, with about one in six developing non-resolving autoimmune disorders. Patients with possible B19V-triggered autoimmune disorders may be distinguished from patients with transient manifestations by more frequent development of arthritis and may share a tendency to develop cutaneous manifestations with patients with pre-existing autoimmunity. When these latter subjects develop symptomatic B19V, milder joint and haematological manifestations could occur compared to patients with new-onset autoimmunity, possibly due to ongoing treatments. Conversely, patients with new-onset autoimmunity often require active immunosuppression with corticosteroids, highlighting the non-negligible burden of morbidity associated with B19V-related immune-mediated manifestations. Accurate baseline patient profiling and adequate monitoring may enable early tackling of B19V-induced autoimmunity and minimise their potential complications.

## Figures and Tables

**Table 1:** Demographic features and comorbidities of the study population upon B19V infection diagnosis.

Variable	Value
Total participants, n	39
Age, years median (IQR)	42 (36–46)
Sex, n (%)	
Male	9 (23%)
Female	30 (77%)
History of Autoimmune Diseases, n (%)	9 (23%)
Hashimoto’s thyroiditis	6 (15%)
Rheumatic disorders	7 (18%)
UCTD	1 (3%)
SLE	3 (8%)
APS	1 (3%)
Arthritis	3 (8%)
Rheumatic fever	2 (5%)
Concomitant immunosuppressive/immunomodulant therapy, n (%)	5 (13%)
Hydroxychloroquine	3 (8%)
Colchicine	1 (3%)
Prednisone	1 (3%)
B19V infection diagnosis	
Epidemiological	7 (18%)
Serological	30 (77%)
IgM	25 (64%)
IgG	4 (10%)
Viral DNA detection	2 (5%)

UCTD: undifferentiated connective tissue disease, SLE: systemic lupus erythematosus, APS: antiphospholipid syndrome.

**Table 2:** Demographic features and clinical manifestations of the study population at B19V infection diagnosis.

Symptoms	Total (n = 39)	No Chronic Autoimmunity (n = 27)	Previous Autoimmune Disease (n = 7)	New Diagnosis (n = 5)
Constitutional	22 (56%)	15 (56%)	5 (71%)	2 (40%)
Fever	19 (49%)	13 (48%)	4 (57%)	2 (40%)
Joint manifestations	32 (82%)	23 (79%)	5 (56%)	4 (80%)
Arthralgia	31 (80%)	23 (85%)	5 (71%)	3 (60%)
Arthritis	10 (26%)	5 (19%) ^^^	1 (14%) ^§^	4 (80%) ^^§^
Skin involvement	26 (67%)	15 (56%) *	7 ^†^ (100%) *	4 (80%)
Rash	17 (44%)	9 (33%) **	6 (86%) **	2 (40%)
Purpura	5 (13%)	4 (15%)	0 (0%)	1 (20%)
Livedo	2 (5%)	1 (4%)	0 (0%)	1 (20%)
Neurological	5 (13%)	3 (11%)	0 (0%)	2 (40%)
Serositis	1 (3%)	0 (0%) ^^^^	0 (0%)	1 (20%) ^^^^
Haematological				
Anaemia	16 (41%)	14 (52%) ***	0 (0%) ***^§§^	2 (40%) ^§§^
Leukopenia	7 (18%)	6 (22%)	0 (0%)	1 (20%)
Thrombocytopenia	1 (3%)	0 (0%)	1 (14%)	0 (0%)
Increased AST/ALT	7 (18%)	6 (22%)	0 (0%)	0 (0%)
Serological				
Hypergammaglobulinemia	9 (23%)	8 (30%)	0 (0%)	0 (0%)
Autoantibodies	17 (44%)	11 (41%)	3 (43%)	3 (60%)
ANA	11 (28%)	6 (22%)	2 (29%)	3 (60%)
Anti-dsDNA	3 (8%)	3 (11%)	0 (0%)	0 (0%)
ANCA	2 (5%)	1 (4%)	0 (0%)	1 (20%)
RF	3 (8%)	2 (7%)	0 (0%)	1 (20%)
aPL	2 (5%)	1 (4%)	0 (0%)	1 (20%)
Cryoglobulins	3 (8%)	3 (11%)	0 (0%)	0 (0%)
Low complement	9 (23%)	8 (30%)	1 (14%)	0 (0%)

^†^ One patient only had pruritus and ecchymosis; *p* values when comparing patients without chronic autoimmunity to those with a previous autoimmune disease: * *p* = 0.028; ** *p* = 0.013; *** *p* = 0.013. *p* values when comparing patients without a history of chronic autoimmunity to those with a new diagnosis of autoimmune disease: ^^^ *p* < 0.001; ^^^^ *p* = 0.018. *p* values when comparing patients with a previous autoimmune disease to those with a new diagnosis of autoimmune disease: ^§^ *p* = 0.010; ^§§^ *p* = 0.039. AST: aspartate aminotransferase; ALT: alanine aminotransferase.

## Data Availability

The data reported in this paper can be shared upon reasonable request to the corresponding author.

## Supplementary Materials

The following supporting information can be downloaded at: https://www.mdpi.com/article/10.3390/v18070774/s1, Figure S1: Percentage distribution of arthralgia and arthritis by joint in patients with B19V infection; Table S1: sensitivity analysis by type of B19 infection diagnosis.
