# Supplementary material for: Distinctive Inflammatory Traits of Parvovirus B19 Infection Associated with Persisting Autoimmunity
Source: Viruses. 2026 Jul 14;18(7):774. doi: 10.3390/v18070774 (PMC13431566; doi:10.3390/v18070774)
Supplement: Supplementary file 1 [file viruses-18-00774-s001.zip › viruses-4389112-supplementary.pdf]

Supplemental material

to

Distinctive inflammatory traits of parvovirus B19 infection associated with persisting autoimmunity

Anna Negri, Maria Chiara Gerardi, Gabriele D. Gallina, Luca Moroni, Antonella Adinolfi, Mona-Rita Yacoub, Nicola Boffini, Marco Lanzillotta, Annalaura Fasiello, Claudia Cordini, Enrica P Bozzolo, Marco Matucci-Cerinic, Oscar Massimiliano Epis, Lorenzo Dagna, Giuseppe A. Ramirez

Supplemental Table S1: sensitivity analysis by type of B19 infection diagnosis

Variables	Total (n=39)	Epidemiological diagnosis (n=7)	Virological diagnosis (n=32)
Demographics			
Sex (female): N (%)	30 (77%)	7 (100%)	23 (72%)
Age (years): median (IQR)	42 (36-46)	42 (36-46)	41 (36-46)
B19V clinical manifestations			
Constitutional	22 (56%)	3 (43%)	19 (59%)
Fever	19 (49%)	2 (29%)	17 (53%)
Joint manifestations			
Arthralgia	31 (80%)	6 (86%)	25 (78%)
Arthritis	10 (26%)	1 (14%)	9 (30%)
Skin involvement	26 (67%)	5 (71%)	21 (66%)
Rash	17 (44%)	4 (57%)	13 (41%)
Purpura	5 (13%)	0 (0%)	5 (16%)
Livedo	2 (5%)	1 (14%)	1 (3%)
Neurological	5 (13%)	1 (14%)	4 (13%)
Serositis	1 (3%)	0 (0%)	1 (3%)
Haematological			
Anemia	16 (41%)	3 (42%)	13 (42%)
Leukopenia	7 (18%)	2 (29%)	5 (17%)
Thrombocytopenia	1 (3%)	0 (0%)	1 (3%)
Increased AST/ALT	7 (18%)	1 (14%)	6 (19%)
Serological			
Hypergammaglobulinemia	9 (23%)	2 (29%)	7 (23%)
Autoantibodies			
ANA	11 (28%)	1 (14%)	10 (40%)
Anti-dsDNA	3 (8%)	0 (0%)	3 (9%)
ANCA	2 (5%)	0 (0%)	2 (6%)
RF	3 (8%)	0 (0%)	3 (9%)
aPL	2 (5%)	0 (0%)	3 (9%)
Cryoglobulins	3 (8%)	1 (14%)	2 (6%)
Low complement	9 (23%)	1 (14%)	8 (38%)

No statistically significant differences were found for categorical variables (by Fisher's exact test) and quantitative variables (by Mann-Whitney U test).

Supplemental Figure S1: Percentage distribution of arthralgia and arthritis by joint in patients with B19V infection

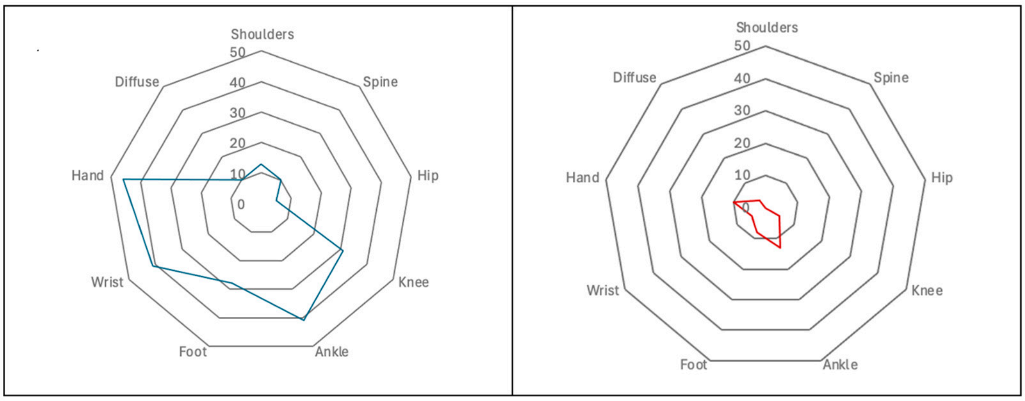

(a) (b)

Radial plot depicting the frequency of involvement of most significant peripheral joint groups in patients with parvovirus B19-related arthralgia (a) and arthritis (b).
